# Neural Network in the Analysis of the MR Signal as an Image Segmentation Tool for the Determination of T_1_ and T_2_ Relaxation Times with Application to Cancer Cell Culture

**DOI:** 10.3390/ijms24021554

**Published:** 2023-01-13

**Authors:** Adrian Truszkiewicz, Dorota Bartusik-Aebisher, Łukasz Wojtas, Grzegorz Cieślar, Aleksandra Kawczyk-Krupka, David Aebisher

**Affiliations:** 1 Department of Photomedicine and Physical Chemistry, Medical College of University of Rzeszów, University of Rzeszów, Warzywna 1A Street, 35-310 Rzeszów, Poland; 2 Department of Biochemistry and General Chemistry, Medical College of University of Rzeszów, University of Rzeszów, Kopisto 2a Street, 35-959 Rzeszów, Poland; 3 Department of Medical Equipment, Provincial Clinical Hospital No. 2, 35-959 Rzeszów, Poland; 4 Department of Internal Medicine, Angiology and Physical Medicine, Center for Laser Diagnostics and Therapy, Faculty of Medical Sciences in Zabrze, Medical University of Silesia, 40-055 Katowice, Poland

**Keywords:** MATLAB, T1, T2, relaxation times, MR

## Abstract

Artificial intelligence has been entering medical research. Today, manufacturers of diagnostic instruments are including algorithms based on neural networks. Neural networks are quickly entering all branches of medical research and beyond. Analyzing the PubMed database from the last 5 years (2017 to 2021), we see that the number of responses to the query “neural network in medicine” exceeds 10,500 papers. Deep learning algorithms are of particular importance in oncology. This paper presents the use of neural networks to analyze the magnetic resonance imaging (MRI) images used to determine MRI relaxometry of the samples. Relaxometry is becoming an increasingly common tool in diagnostics. The aim of this work was to optimize the processing time of DICOM images by using a neural network implemented in the MATLAB package by The MathWorks with the patternnet function. The application of a neural network helps to eliminate spaces in which there are no objects with characteristics matching the phenomenon of longitudinal or transverse MRI relaxation. The result of this work is the elimination of aerated spaces in MRI images. The whole algorithm was implemented as an application in the MATLAB package.

## 1. Introduction

Artificial neural networks have a number of possibilities that are worth exploiting in medical research. From a practical point of view, neural networks used in magnetic resonance imaging (MRI) research are based on operations on vectors and matrices. In particular, herein we used the MATLAB package by The MathWorks for matrices calculations. The mechanisms focused on matrices are among the most efficient in neural networks.

It should be noted that MRI hardware solutions involve the implementation of neural networks to integrated circuits. These solutions, although very efficient, are not suitable for research studies such as the present one. However, they will work well in targeted and optimized applications.

MRI is a leading image modality in daily clinical research. The only concern is the description of the obtained images. There is a shortage of radiologists, so the waiting time for a description can be extended. When an experienced radiologist is required for each MRI diagnosis, the assessment of neural network can relieve specialists and become routine tests [1,2,3].

Artificial intelligence (AI) presents tremendous progress in the field of MRI. Promising approaches include deep learning methods for reconstructing MRI data and generating high-resolution data from low-resolution data. Preliminary studies show that the state-of-the-art technique can generalize many different anatomical areas and achieve comparable diagnostic accuracy with conventional methods. This article discusses state-of-the-art methods, clinical application considerations, and then future prospects in the MRI field.

Today, relaxometry is an excellent clinical tool for conducting research and gaining a diagnosis in areas such as brain and spine tumor [4], pancreas [5], liver [6], human limbic white matter [7], cerebral white matter [8], Parkinson’s disease [9], Alzheimer’s disease [10], and many others. The number of excellent systematic reviews of methodological methods is increasing [11,12,13,14].

MRI systems are devices containing analog–digital paths that process signals with low or very low values. They have a number of blocks corresponding to generation, signal amplification, or its A/C processing. The input stages of the coils reject and transmit signals at very low levels. The high level of automation of the processing systems means that the amplifying stages can perform switching of the signal amplification stage in a completely autonomous manner. This state of affairs means that the use of standard research protocols may result in different values of signal intensity in individual sequences. In T_1_ and T_2_ measurements, the signal intensity is taken as the basic factor for creating graphs and approximating measurement points. Elimination of such equipment errors is difficult and requires excellent knowledge of the operation of the systems, support provided by the device manufacturer may also be helpful.

A sample placed in a magnetic field has a magnetization value defined as M0. The rotation of a proton can be compared to the rotation of a gyroscope. The frequency of these rotations depends on the value of the magnetic field and the previously mentioned gyromagnetic coefficient. For 1.5 Tesla induction, the resonant frequency is 63.86621838 MHz, while for 3 Tesla is 127.73243676 MHz. The equation describing the dependence of the resonant frequency on the value of the magnetic field is as follows:

(1) $$f_{0}=\frac{1}{2\pi }\gamma B_{0}$$

**Figure 1:**
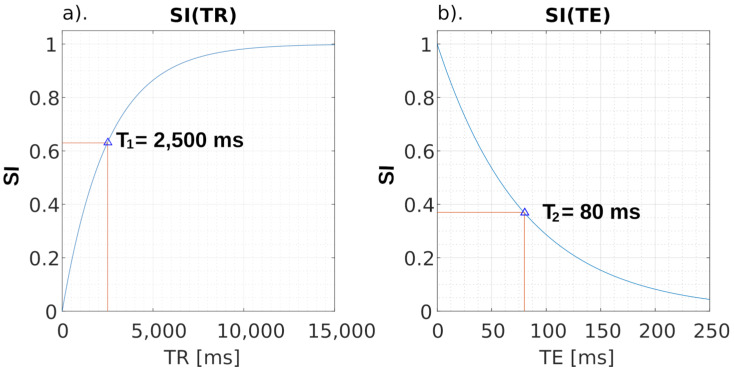
The graphs show the shape of the signal intensity for (**a**) longitudinal relaxation time T_1_ and (**b**) transverse relaxation time T_2_. For time T_1_, it is a graph of SI as a function of repetition time TR, while for time T_2_, the graph presents SI as a function of echo time TE. The presented graphs are digitally generated waveforms to illustrate the phenomena themselves and the relationships between them. In the drawings, the points at which T_1_ and T_2_ were determined are marked with a triangle sign, i.e., these are the times for which SI is 63% and 37% of the maximum value of the signal, respectively.

As a result of the interaction between the electromagnetic wave with resonant frequency, the magnetization vector deviates from the position parallel to the field lines B0, and the value of this deviation depends on the intensity of this wave. After its action ceases, the equilibrium is restored–the time needed to return to the steady state before excitation is called the relaxation time. It should be added that relaxation is longitudinal T_1_ and transverse T_2_ time (Figure 1).

The relaxation time T_1_ is the time needed for the longitudinal magnetization in the Z axis to return to 63% of its original state. This relaxation is referred to as a spin-lattice. The reason is that in the process of return energy is transferred to the environment. The speed of the process depends on the force with which the protons interact with the environment and is the faster the more macromolecules there are in the examined tissue. The longest T_1_ times occur for tissues with the highest water content and the lowest content of macromolecules [15]. Longitudinal relaxation time depends on, e.g., temperature [16] and viscosity of the environment [17].

The time T_2_ is related to the phase of the spins. The phenomenon of phase coherence decay depends on the tested object itself. It also depends on the parameters of the magnetic resonance system itself. In particular, the parameter is affected by the uniformity of the B_0_ field. The imperfection of the homogeneity of the field causes the individual nuclei to be in different magnetic fields. These are very small differences, but enough for the protons to have different resonant frequencies. This leads to dephasing of the system and decay of the transverse component of the magnetic field. Another reason for the disappearance of the transverse component is the properties of the sample itself. For these reasons, transverse relaxation is referred to as spin-spin relaxation.

The signal that is received in the MRI system can be described by the relationship:

(2) $$SI\approx PD\left(1-e^{\frac{-TR}{T1}}\right)e^{\frac{-TE}{T2}}$$

where: SI–signal intensity, PD–tissue proton density, T_1_–longitudinal tissue relaxation time, T_2_–transverse tissue relaxation time, TE–echo time, TR–repetition time.

Equation (2) describes in a relatively simple way the intensity of the signal that is received by the receiving coils. Analyzing this relationship, two of the parameters contained in it depend on the operator and the system capabilities (TE, TR), and three are purely material relationships that are the property of the tested object itself. Medical imaging in T_1_-weighted and T_2_-weighted images consists in recording the signal at fixed echo and repetition times. This includes choosing their values allows one to show the assumed cross-section of the object in a better or worse way.

Equation (2) also allows to determine the relaxation times of the tested objects. Analyzing it, we can see that by selecting the appropriate values of the TE and TR parameters, we can eliminate the dependence with respect to T_1_ or T_2_ from the equation, and thus from the picture. This leads, in a simple way, to determining the repetition times.

In order to determine the T_1_ time, the echo time value should be set as low as possible. Then, the term exp((−TE)/T_2_) will be close to “1”, and Equation (1) can be written as

(3) $$\underset{TE\to 0}{\lim}\left(PD\left(1-e^{\frac{-TR}{T1}}\right)e^{\frac{-TE}{T2}}\right)=PD\left(1-e^{\frac{-TR}{T1}}\right)$$

For the search of accuracy, the term containing the T_2_ time will be a constant value slightly less than 1 and it will somehow scale the SI value. In order to determine the T1 time, the tested layer should be acquired at different TR times, starting from small values up to the maximum possible to be set in the camera. It should be added here that clinical scanners have a TR value that can be set at 15,000 ms. In practice, data should be collected for a maximum TR time equal to or greater than 5 times the value of the expected T_1_ time. Reducing the maximum TR time will increase the error associated with the determination of T_1_ time.

Then, on the SI(TR) chart, determine for what time the SI reaches the value of 63%. This value corresponds to the point where TR = T1:

(4) $${\left(1-e^{\frac{-TR}{T1}}\right)}_{TR=T1}\approx 0.63212\approx 63\%$$

In a similar way, we can present the possibility of determining the transverse relaxation time–T_2_.

Assuming then TR to be as large as possible, the term exp((−TR)/T_1_) will be eliminated–for long TR times, it will assume a value close to “0”.

(5) $$\underset{TR\to \infty }{\lim}\left(PD\left(1-e^{\frac{-TR}{T1}}\right)e^{\frac{-TE}{T2}}\right)=PDe^{\frac{-TE}{T2}}$$

Also in this case, the determination of the T_2_ time consists in determining the point on the time axis–TE, at which the IS decreases to the value of 37%. Equation (5) will take this value only when TE = T_2_, i.e., the exponent is “−1”.

(6) $${\left(e^{\frac{-TE}{T2}}\right)}_{TE=T2}\approx 0.36788\approx 37\%$$

For the accuracy, it should be added that it is also possible to record images related to proton density–PD. Then, TR should be set as a long time–many times longer than T_1_ and TE as a short time–many times shorter than T_2_. Then, the T_1_-dependent term will be zeroed, while the T_2_-dependent term will slightly influence the PD intensity. A large number of measurement points is recommended to minimize approximation errors. However, this extends the test time.

Artificial intelligence is increasingly involved in creating an image based on numerical data. This requires powerful applications as well as the computer systems themselves. Neural networks in medicine can be found in the vast majority of specialties. One of the first places seems to be attributed to imaging diagnostics.

This work presents the use of a neural network for MRI data analysis, on the basis of the distribution of times relaxation times T_1_ and T_2_. This article does not describe the classic approach to image segmentation based on an algorithm related to the pixel intensity in a single image.

The aim of the work was to present the use of neural networks for the analysis of data obtained during the MRI examination. The application presented here will not serve the traditionally understood segmentation, but an approach to the analysis of data from the measurements of T_1_ and T_2_ relaxation times by the method of saturation recovery (SR) will be shown. Sequences of images and their relationship to each other will be analyzed, and not, as in the traditional image recognition technique, the analysis of pixels adjacent to one plane.

In determining the relaxation time, from a few to a dozen or so DICOM 3.0 images are taken into account. These images are different as a result of changing the repetition time (TR) and echo (TE) parameters.

Artificial neural networks, through their properties, can learn, and thus have a number of possibilities that are worth using. From a practical point of view in technology, neural networks are for the most part a creation based on operations on vectors and matrices. The implemented mechanisms focused on working with matrices are among the most efficient.

It should be noted that there are also hardware solutions involving the implementation of neural networks to integrated circuits. However, these solutions, although very efficient, are not suitable for research work such as the present one. They will work well in targeted, optimized, and reproduction applications.

**Figure 2:**
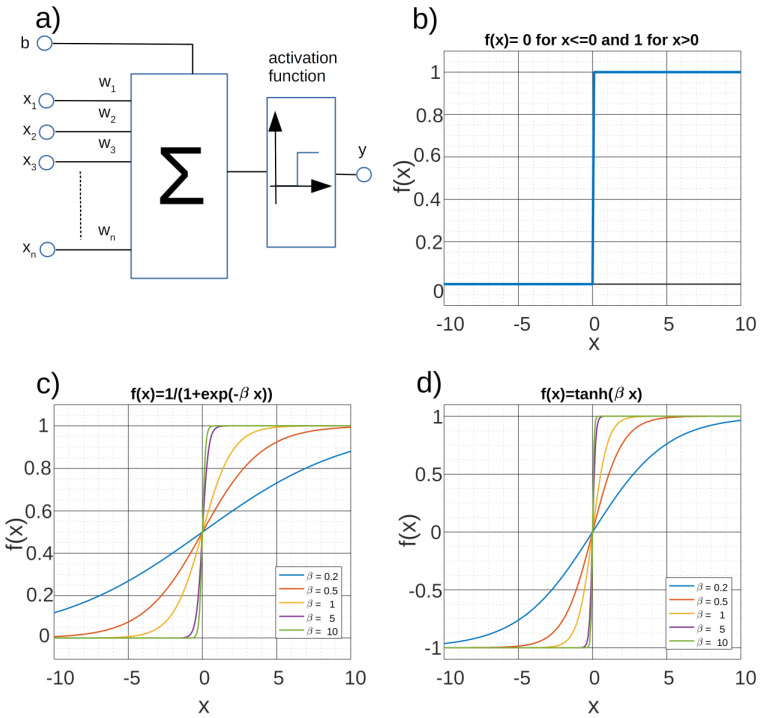
(**a**) Basic scheme of a single neuron (perceptron basic diagram); (**b**) the course of the function response taking only two values–[0, 1], the function f(x) is called the Heaviside step function; (**c**) graph of the unipolar sigmoidal function response of the neuron–the values accepted by the activation function are in the range <0.1>, the function of f(x) is known as “logistic growth function’ and β represents the “logic growth rate”; (**d**) graph of the neuron’s bipolar response–the values of the activation function are in the range <−1.1>. In (**b**,**c**), the slope of the curve depends on the parameter B (beta), increasing the value of this parameter brings the graph closer to a step function. Both of these functions are continuous functions.

The basic element of any neural network is a single neuron. Figure 2 shows schematic diagram. Symbols: x1... xn–network inputs, w1...wn–weights of individual inputs, y–output. For clarification purposes, each input has its own “amplification factor” called weight by which it is multiplied. In general, the output signal can be described by the relationship:

(7) $$y=f\left(b+\sum_{i=1}^{n}\left(x_{i}\left(t\right)\cdot w_{i}\right)\right)$$

The expression which is the argument of the function f is a sum signal. It amounts to:

(8) $$u=b+\sum_{i=1}^{n}\left(x_{i}\left(t\right)\cdot w_{i}\right)$$

(9) $$\left(u\right)=\{\begin{array}{c} 0\;for\;x\leq 0\\ 1\;for\;x>0 \end{array}$$

It is a step function, which takes only two values: 0 and 1. Other continuous activation functions are functions sigmoidal unipolar:

(10) $$f\left(x\right)=\frac{1}{1+e^{-\beta x}}$$

and bipolar:

(11) f(x)=tanh(βx)

The value of the parameter β is selected by the user and affects the shape of the activation function. Increasing the value of this parameter increases the steepness of the function. For β→∞ these functions become step functions.

A multilayer network was used in this work. It is a variant of neural networks containing, in addition to the input and output layers, hidden layers of neurons.

This work describes the application of a unidirectional, multilayer neural network with a sigmoidal activation function. The flow of information in such a network is one-way–from input to output. The mathematical description of such a network is relatively simple. Similarly, the learning methods are uncomplicated. This type of neural network is most often trained in the “with a teacher” mode. In the learning mode, it is therefore necessary to provide the inputs of the network with a training set and the required response. This is also the case with this work. The training set is a sequence of slices of medical images saved in the DICOM 3.0 standard from two regions. The first region is the sample region, while the second region is the aerated noise region. The first region as a response has a value of “1” for each pixel, while the response for noise is a value of “0”. The sequence for training the neural network comes from the same image that will be segmented. The use of such a method allows the training to take into account the noise inherent in the MRI image and coming from the currently used system and sample. This approach to learning is not often used; however, the segmentation process gives very good results, which, combined with the short time necessary for learning, is optimal. The network learning operation for a sample containing 200 training vectors takes 0.5 s. This time is obtained in a PC system based on an i5 processor, 16 GB RAM, 3.4 GHz, 500 GB SSD.

## 2. Results

**Figure 3:**
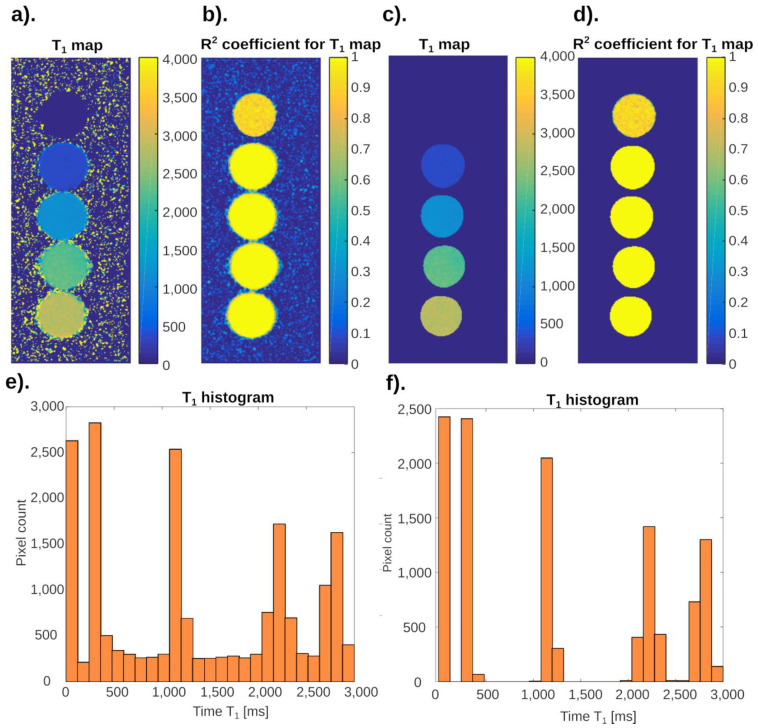
Distribution of T_1_ relaxation time values for the tested phantom, the color of each pixel corresponds to the T_1_ value that was measured for a specific pixel in a series of DICOM images: (**a**) the distribution of T_1_ times before the analysis with the neural network; (**b**) the distribution of fit coefficient R^2^ values before the neural network analysis; (**c**) the distribution of T_1_ times after the analysis with the neural network; (**e**) histograms of T_1_ times from images before the analysis with the neural network; (**f**) histograms of T_1_ times from images after the analysis with the neural network.

Figure 3 presents the results of processing the examined phantom with two methods, namely without the use of neural networks and with their use. The image shows differences in the aerated area, because in this part the neural analysis showed noise, so the pixels forming this area were automatically assigned the value “0”. For calculations, “neural networks qualified” only the areas of the phantom.

Figure 3a,c shows the images of the phantom differ mainly in the aerated area–the place where there is no phantom. This was also shown in two histograms, Figure 10e,f. Comparing the values in the histograms, the lack of noise is clearly visible. However, it seems more important to reduce the height of individual peaks. This is due to the fact that their height is influenced by pixels from the space outside the phantom. This, in turn, has a positive effect on the credibility of the results when quantifying based on the analysis of histograms. In addition, the areas bordering the phantom and the test tube, or the test tube itself, were cut out of the analysis area.

Also analyzing the time needed to perform the computational task, it should be stated that it was significantly shortened, compared to the time needed to perform the calculations without the use of neural network analysis. Although the results related to the processing time analysis can be considered biased due to the size of the analyzed images, the computer system used and the imperfections of the algorithm, they show the potential of advanced analysis using neural networks. The analysis of images without the neural network took about 35 min, while omitting the pixels that are noise using the neural network allowed to reduce the time to less than 6 min. These times are sometimes relatively long. Today’s sophisticated algorithms on specialized workstations in combination with appropriate data acquisition methods allow for making such calculations in much shorter times. Nevertheless, the use of a neural network allows to improve the situation in this respect. It should be added that this ratio is fully dependent on the ratio of airspace pixels to phantom space pixels.

In this case, the preparation of the data and training of the neural network takes less than 5 s, so it can be omitted in the estimation of the time reduction.

**Figure 4:**
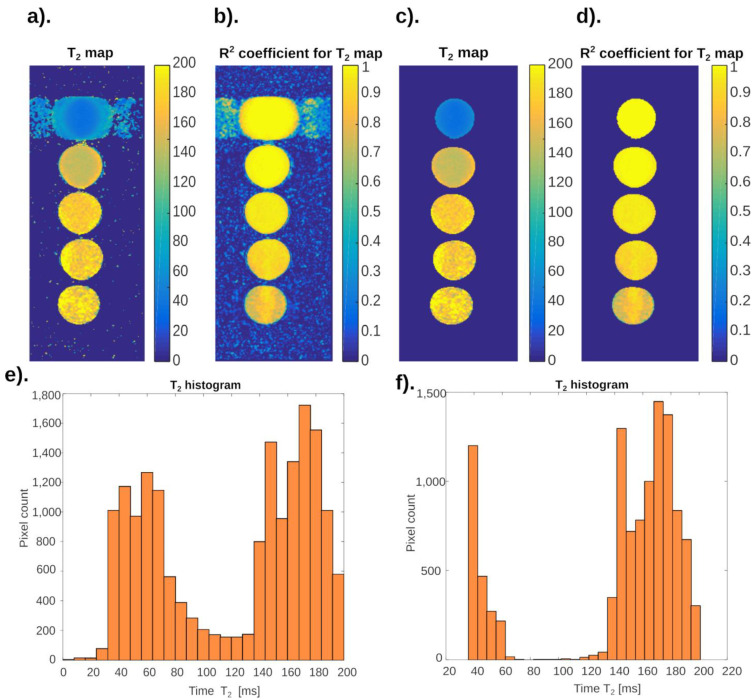
Distribution of T_2_ time values for the tested phantom, the color of each pixel corresponds to the value of the longitudinal relaxation time that was measured for a specific pixel in a series of DICOM images: (a) phantom maps showing the distribution of T_2_ times before the neural network analysis; (**b**) phantom maps showing the distribution of fit coefficient R^2^ values before the neural network analysis; (**c**) phantom maps showing the distribution of T_2_ times after the neural network analysis; (**d**) phantom maps showing the distribution of fit coefficient R^2^ values after the neural network analysis; (**e**) histograms of longitudinal relaxation times from images before the neural network analysis; (**f**) histograms of longitudinal relaxation times from images after the neural network analysis.

Figure 4 shows an image analysis of the transverse relaxation time T_2_ mapping. As in the previous case, there was a significant improvement in the quality of the image obtained. Artifacts at the sample with the shortest T_2_ time have been removed. Noise has also been eliminated, and the quality of the histogram has improved.

Repeated attempts to choose the place from which the samples for training the neural network are taken have shown that it is advisable that these are areas with short T_2_ times. R_2_ is the coefficient of determination in statistics to assess the quality of analysis of image parameters.

Each of the T_1_ or T_2_ mapping images consists of two parts. The first is an image that is a map of the distribution of relaxation times and the second is an image of the distribution of the R^2^ coefficient. The R^2^ coefficient is a measure of the quality of the model fit–in this case, a measure of the fit of the approximating function to the dataset. Each set of data in question is the signal intensity values for a given pixel read from all DICOM images. When analyzing the R^2^ image, it should be assumed that its selected pixel represents a measure of the quality of matching to the corresponding pixel in the images being the T_1_ and T_2_ maps. Thus, these images have the advantage over a single numerical value that they show exactly where the approximation curve is the best fit to the data and where it is worse. The data analysis proposed in this article consists in displaying statistics in relation to individual pixels of the image. This is essential in a situation where images containing different tissues that do not have the same T_1_ or T_2_ times will be analyzed. Determining then any statistics, e.g., the average or standard deviation from a specific image surface, will be a substantive error because the value of the statistics will be influenced by different values resulting from the properties of the tissues and not the acquisition process, noise, or other interfering factors.

The value of the coefficient of determination is obtained as a result of the approximation functions implemented in MATLAB. It is defined:

(12) $$R^{2}=\frac{SSR}{SST}=1-\frac{SSE}{SST}$$

where: SSE is the sum of the squares of the errors, SSR is the sum of the squares of the regression, SST is the sum of the total squares, $y_{i}$ is i-th observation of variable y, $\hat{y_{i}}$ is theoretical value of the variable explained on the basis of the model and $\bar{y}$ is the arithmetic mean of the empirical values of the dependent variable.

(13) $$SST=\overset{n}{\underset{i=1}{\sum }}{\left(y_{i}-\bar{y}\right)}^{2\;},\;\;\;SSE=\overset{n}{\underset{i=1}{\sum }}{\left(y_{i}-\hat{y_{i}}\right)}^{2\;},\;\;\;SSR=\overset{n}{\underset{i=1}{\sum }}{\left(\hat{y_{i}}-\bar{y}\right)}^{2\;}\;,\;\;\;$$

For accuracy, it should be added that the coefficient of determination itself may take values close to “1”, even with a poorly selected model. In the case of this article, there is no such danger because the mathematical description of the phenomenon is known and has been implemented as an approximation function.

**Figure 5:**
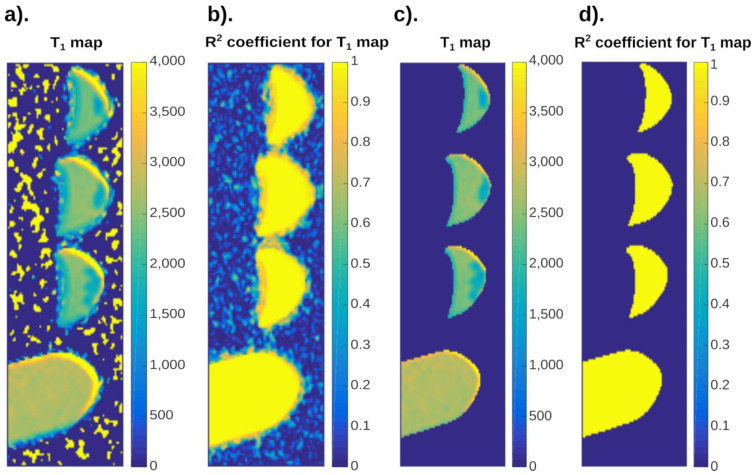
Practical application of neuronal analysis in cell cultures. Distribution of T_1_ values for the MCF-7 cell culture: (**a**) maps showing the distribution of T_1_ times before the neural network analysis; (**b**) phantom maps showing the distribution of fit coefficient R^2^ values before the neural network analysis; (**c**) maps showing the distribution of T_1_ times after the neural network analysis; (**d**) phantom maps showing the distribution of fit coefficient R^2^ values after the neural network analysis.

Figure 5 is a practical example of neural networks. The figures below show the result of T_1_ mapping of MCF-7 breast cancer cell cultures. These cells are located in the lower part of the Eppendorf and are clearly demarcated from the culture fluid (for clarification, it should be added that the images have been “turned” to a vertical position). Details related to the culture of these cells are included in the paper [18].

Noise reduction is clearly visible. The signal from the test tubes, which is an artifact, was also largely eliminated.

Figure 5a shows the cell mapping results. In the aerated space, the places where the approximation algorithm calculated the longitudinal relaxation time T_1_ were marked in red. The specified value is well above 4000 ms. Such a state of affairs is impossible; however, the series of signal intensity vs. time TR data together with noise resulted in an incorrect determination of the airspace relaxation time. The neural network trained on the image successfully rejected these data as incorrect. The data showing the edge of the test tubes–red color on the edge of the test object–were also rejected. The neural network, while segmenting the data, also rejected the upper part of the culture fluid in which the cells were immersed. The fluid–air interface has a distorted relaxation time estimation and is an artifact that has been successfully removed. The phenomenon of partial volume and its impact on the obtained images has been discussed in [19]. There, on the example of a deliberately phantom, changes in the T_1_ time values were shown when the 7 mm wide layer covered by the measurement contained both water and gel. The water–gel ratio as a function of the layer length was variable, which allowed to visualize the effect of partial volume.

**Figure 6:**
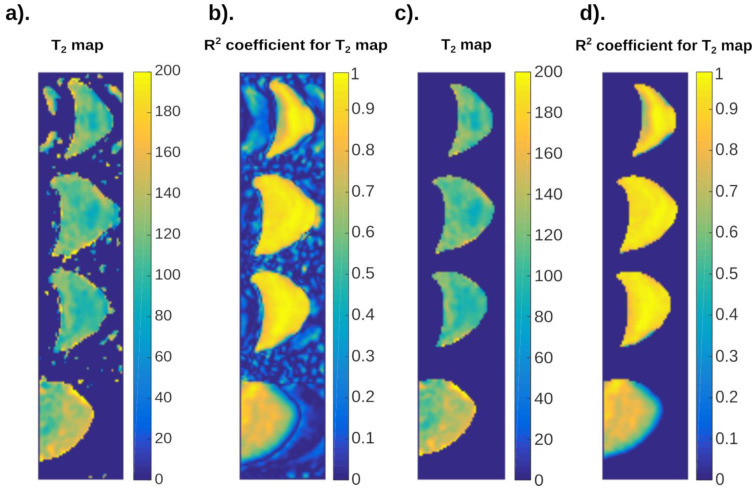
Practical application of neuronal analysis in cell cultures. Distribution of T_2_ values for the MCF-7 cell culture: (**a**) Map showing the distribution of T_2_ times before the neural network analysis; (**b**) phantom maps showing the distribution of fit coefficient R^2^ values before and after the neural network analysis; (**c**) Map showing the distribution of T_2_ times after the neural network analysis; (**d**) phantom maps showing the distribution of fit coefficient R^2^ values before and after the neural network analysis. Noise reduction is clearly visible.

Figure 6 presents the results of the analysis of T_2_ with neural networks as well as without them for the MCF-7 cell culture. As with the T_1_, there was a reduction in artifacts and noise. The change in the image scale is caused by a different choice of ROI for analysis. Changing the image scale does not adversely affect the visualization of the neural network.

**Figure 7:**
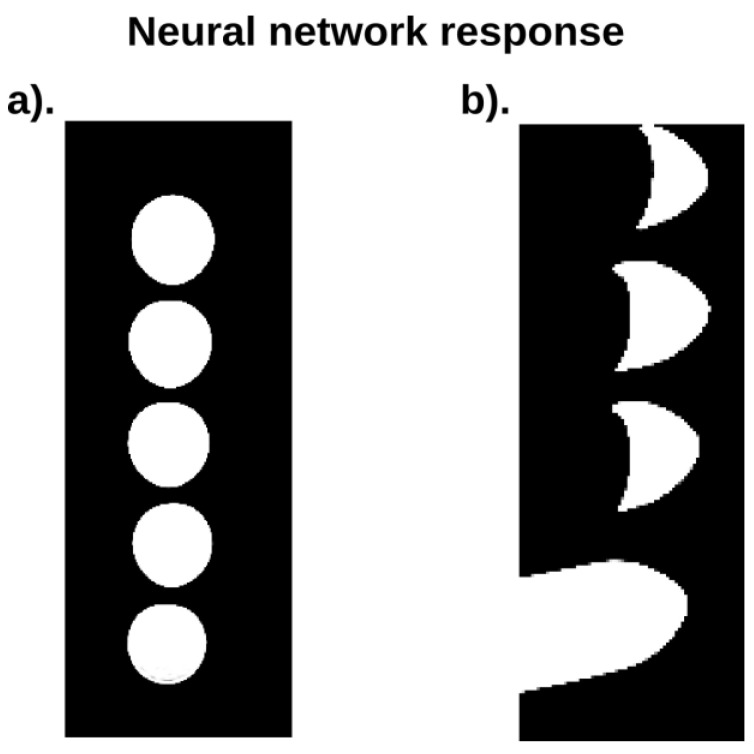
(**a**) presents the “response” of the neural network that was obtained as a result of the analysis of DICOM image sets for the T_1_ longitudinal relaxation time in phantom consisting of 5 test tubes; (**b**) presents the “response” of the neural network that was obtained as a result of the analysis of DICOM image sets for the T_1_ longitudinal relaxation time in MCF-7 cell culture. The task of the neural network was to classify the data into two categories. The first is the category of the area whose MR characteristics correspond to the resonance characteristics of magnetic resonance phenomena–white area, while the second is the area whose behavior does not correspond to MR phenomena–black area. On the basis of this answer, the aerated areas were not analyzed, which allowed to reduce the time needed for data processing and thus to optimize it.

Figure 7 shows the response of the neural network. In the images, black color shows “0”, i.e., places that artificial intelligence algorithms considered noise. The white color represents places that have been classified as “1”, i.e., objects that are phantoms or cell cultures.

## 3. Discussion

A neural network is a computing system with interconnected nodes that work much like neurons in the human brain. Using algorithms, this network can recognize hidden patterns and correlations in raw data, cluster and classify it, and continuously learn and improve [18]. Recently, neural networks are becoming common tool in the hands of engineers supporting the medical fields. It is known that neural networks exceed quantitative and qualitative assessments of imaging. The processing of images with neural networks has become useful in medical diagnostics [19]. Some examples include colon examination in patients with a potential risk of colorectal cancer [20]. The risk of osteoporosis was predicted with great accuracy using AI [21]. The detection of breast asymmetry and classification of calcifications in breast was investigated with neural network [22]. Other studies [23,24,25,26,27] have found neural network useful in mammography screening. MRI with together with the neural network was used in the analysis of the liver [28], myocardium [29,30,31,32], and breast [33]. The possibility to use the neural network for MRI data is helpful tool in medical research. Thus, in clinical concepts, it can be useful to recognize the margin, texture, speculation, and lobulation of the MRI image. Quantitative and qualitative evolution of MRI images with the use of neural network can help in discovering new imaging biomarkers. The algorithms of neural network are based on training possibility to perform the multiple trials. The proposed scheme of operation allows for the variability in the characteristics of imaging systems. Interpretability of neural network approaches for MRI is important for clinical trust and for troubleshooting systems [34]. In the literature, work can be found that uses the concept of artificially generated negative data to form decision using a multilayer perceptron [35]. A few models of neural networks are known, e.g., Convolutional Neural Networks (CNNs) [36,37], Recurrent Neural Networks (RNNs) [38], Generative Adversarial Networks (GANs) [39], and Quantitative Susceptibility Mapping (QSM) [40,41]. Particularly, CNNs were most widely used for MRI and other image processing applications. MRI data are typically input as two dimensional (2D) or three dimensional (3D) and procced the arrays of pixels with neural network [42]. Moreover, deep learning methods were used in a model coupled with digital mammographic imaging to evaluate the classification between breast density categories. The next example such as RNNs contain data feedback loops. This feedback serves as a type of “memory” allowing them to use recent outputs as updated inputs for subsequent calculations. Loss of spatial resolution can be overcome by inserting skip connections between the sides to pass through important details to the output image [29]. GANs consist of two competing components: (1) the Generator, a deconvolutional network that uses random noise and interpolation to generate “fake” but realistic-looking images, and (2) the Discriminator, a conventional CNN previously trained with supervised learning to identify real images at a certain level of accuracy. Moreover, QSM is a growing field of research in MRI, aiming to noninvasively estimate the magnetic susceptibility of biological tissue. Mapping the signals back to known tissue parameters (T_1_, T_2_, and proton density) is then a rather difficult inverse problem [41,43]. Estimation of noise and image denoising in MRI has been an important field of research for many years, employing a plethora of methods.

The latest studies have shown that deep learning and radiomics based on hepatic CT and MR imaging have potential application value in the diagnosis, treatment evaluation, and prognosis prediction of common liver diseases [44,45].

## 4. Methods

**Figure 8:**
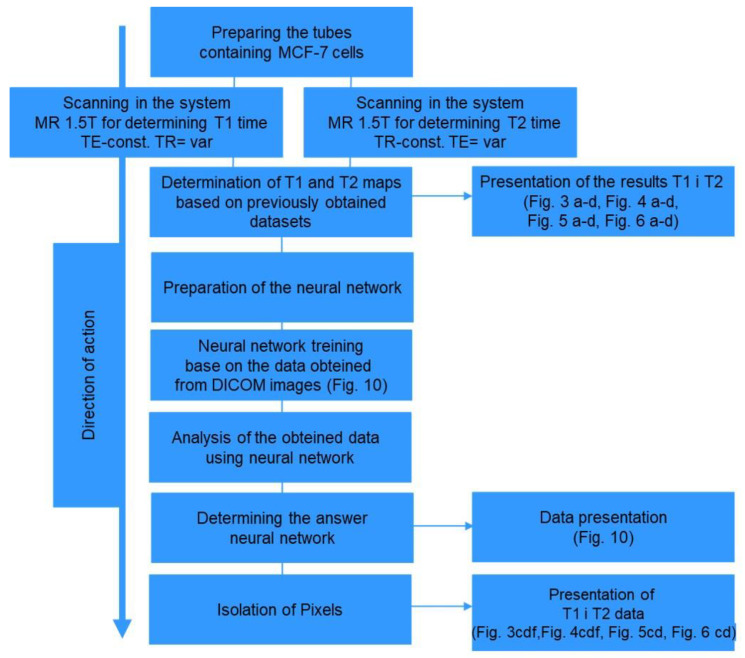
The algorithm of performed steps.

The algorithm (Figure 8) describes the sequence of operations necessary to apply neural networks in the analysis of the MRI signal as an image segmentation tool for the determination of T_1_ and T_2_ relaxation times with application to cancer cell culture.

The first step is the preparation of cell culture samples. In this case, it was MCF-7 breast cancer cells.

The next step was to scan them in the MR OPTIMA 360 1.5 Tesla system. The scanning included the acquisition of data necessary to develop images that are maps of the distribution of T_1_ and T_2_ relaxation times. The maps made it possible to proceed to the next step, namely the calculation of the appropriate relaxation times for each pixel of the image, both transverse and longitudinal.

In order to develop the optimization method, a neural network was created in MATLAB, whose task was to analyze the data collected earlier. Each of them also requires a training process. It was the next step in action. It was based on data from the images that were to be analyzed. It was here that it became necessary with human participation to determine which part of the image is noise and which is not. On this basis, the neural network algorithms trained the network. The neural network was matched to the data acquired during the acquisition. The number of inputs was equal to the number of images making up the number of measurement points taken into account in the approximation process. The output of the network was one neuron with a unipolar response function. After the training was completed, the raw data were processed using the artificial intelligence method. This pattern recognition method classified raw pixel-by-pixel DICOM images into two categories. One of them, noise, was assigned a value close to “0” and the other, a non-noise value, close to “1”.

Based on the classification, the data were re-analyzed to determine the time gain. As a result of a series of tests carried out in the analyzed space, it was possible to reduce the time from approximately 35 min for full analysis up to approximately 6 min using artificial intelligence methods. Although it should be added that this result may seem biased and that this time depends on the ratio of the aerated area and the area depicting artifacts to the area corresponding to the areas of the phantom or cell culture, it must be said without doubt that the neural network allowed to significantly reduce the time necessary for calculations. The work was carried out using a PC class system equipped with an i5 processor, 16 GB RAM, 250GB SSD. For the tests, the authors deliberately chose a solution based on a middle-class system to show that the use of methods related to neural networks allows for significant optimization of work. The use of faster systems, in particular those equipped with graphics cards with CUDA (Compute Unified Device Architecture) options, would allow for further improvement in this area; however, the idea of this work was to show the possibilities related to neural networks in average conditions.

The final stage in the algorithm was the presentation of data both in the form of maps of the T_1_ and T_2_ distributions as well as histograms. It is the frequency analysis shown in the histogram charts that says the most about the qualitative gain of the work. Note that the height of the histogram is affected by pixels regardless of their position in the image. So the height of the graphs is also modified by pixels in the noise-aerated area. The network analysis removed the noise and thus limited its influence on the histogram.

For the experiment, four test tubes (No. 1–4) containing an aqueous CuSO_4_ solution of various concentrations and one sample of distilled water were prepared.

Test tube No. 1 contained 10 mL of distilled water and 0.1079 g of copper sulphate with a molar mass of 159.609 g/mol was added. Each subsequent test tube was prepared in the same way, namely 1 mL of the solution was taken from the previous test tube and supplemented with 9 mL of distilled water. These four solutions were significantly different in concentration to vary the T_1_ and T_2_ times. The T_1_ measured with FSE sequence was used in the study. The TE time was 20 ms, while the TR times were, respectively: 40, 50, 60, 78, 80, 100, 120, 140, 200, 240, 300, 400, 500, 600, 700, 800, 1000, 1500, 2000, 3000, 5000, 10,000, 15,000 ms [18]. The increased number of measurement points is necessary to determine a significant range of T_1_ times that occurred in the study. Their large amounts have a positive effect on the process of approximation of the obtained results. In this case, there is no need to minimize the examination time, as is the case in the examination of patients.

**Figure 9:**
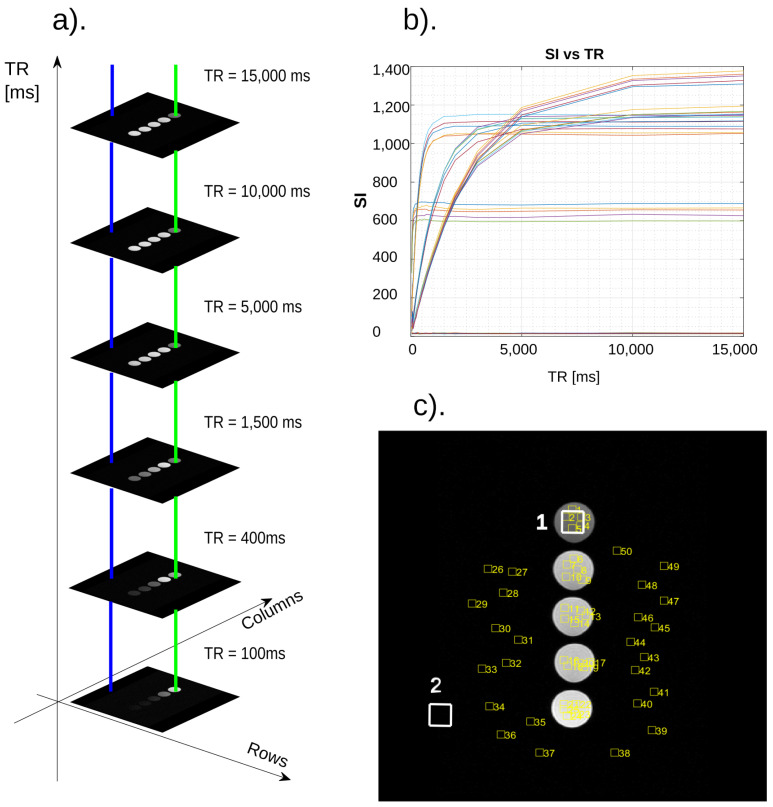
Panels (**a**–**c**) shows the method of acquiring data from a series of DICOM images for the stage of training the neural network. (**a**) Two colored lines passing through the image areas visually show where and how the data are collected. (**b**) The SI graphs in the TR function show the waveforms of the function describing the longitudinal relaxation time T_1_–these are real data obtained as a result of scanning the samples. (**c**) Selected scan–white markings refer to the places of data download for neural network training, yellow numbers are the points shown in (**b**). For the sake of accuracy, it should be added that the graphs of the noise values are located close to the horizontal axis, which makes them slightly emphasized in the scale of the graph.

The materials for this article were images saved in the medical DICOM 3.0 standard. They came from the phantom study described above. Every neural network requires a learning process. This process takes place with the use of data whose nature is known. In this case, data were collected from two areas of the image. The first area is the facility area, while the second is the aerated area. For each series, data were collected from this scan and from the same location. This is shown in Figure 9a where sample acquisition sites are marked with colors, while in Figure 9c, acquisition areas are marked with large numbers. Shown in Figure 9c, the areas numbered from 1 to 50 are the places from which the data for the graph in Figure 9b came. Analyzing these data, it can be seen that the curves coming from individual places, which are images of the phantom, differ in shape. Basically, we are still dealing with an exponential function; however, it differs depending on the value of time T_1_. Moreover, the maximum SI values from individual tubes are characterized by high variability. Waveforms originating from the aerated area are located close to the horizontal axis. Noteworthy is the fact that individual test tubes differ in maximum intensity.

The task of the neural network was to segment the image in such a way that only the area of the tested object remained for calculations. Downloaded data are slices of images with a side of 10 pixels. As a result of this operation, two sets of data were obtained, which from the point of view of the MATLAB program, were matrices with dimensions [x,y,z], where x and y are the dimensions of the sample, while z is the number of analyzed series that were made during the study to determine individual times T_1_ and T_2_. Each data vector–as it was described earlier–was defined with a response value of “0” for the aerated area or “1” for pixels depicting the test object.

**Figure 10:**
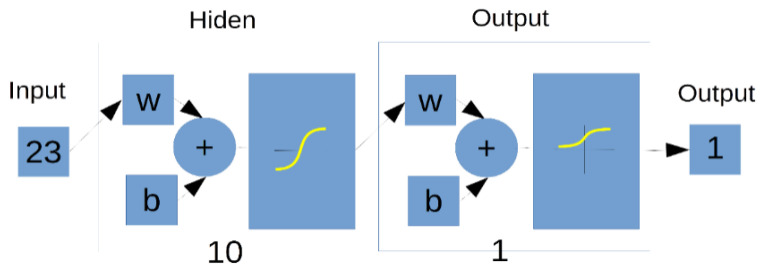
The structure of the neural network as it was implemented for the needs of the analysis. The number of inputs was 23, the number of neurons in the hidden layer, 10. The network contains one unipolar response type output.

Figure 10 presents the structure of the neural network that was created in MATLAB, taking into account the data about the study and the number of acquisition points. In this case, it has 23 inputs, 10 neurons in the hidden layer, and one neuron in the output layer. The activation functions for the hidden neurons and the output neuron are bipolar functions and unipolar functions, respectively.

The listing below shows the MATLAB neural network function

function net=dicom_neural_net(DataDoNet,Resp);
% -------------------------------------------------- ----------
The %function creates a neural network and trains it
% using input data
% Function output parameter:
% net–neural network
%Function input parameters:
%DaneDoNet–input data being a matrix with dimension N * M x S where N and M are
% dimensions in pixels are the dimensions of the area retrieved from the files
% DICOM, while S is the amount of images collected and present
% basis for determining the T1 curve
% Answer–response vector with dimension N*M
% -------------------------------------------------- ----------
net = patternnet(10);
net = train(net,double(DataDoNet),double(Reply));
view(net);

## 5. Conclusions

Neural network used in the analysis of the MR signal as an image segmentation tool for the determination of T_1_ and T_2_ relaxation times with application to cancer cell culture were based on training data. In this paper, neural networks can learn on their own and improve the accuracy of an image. In the phantom samples, changes in the T_1_ time values were shown when the 7 mm wide layer covered by the measurement contained both water and gel. The water–gel ratio as a function of the layer length was variable, which allowed to visualize the effect of partial volume. The changes in the images were influenced in changing the image visualization of the neural network.

By carrying out this research and using a neural network, we were able to carry out much faster analysis than when they are performed manually by researchers.

## Data Availability

Not applicable.

## Abbreviations

CT, computed tomography; SI, signal intensity; MCF-7, breast cancer tumor cells, MRI, magnetic resonance imaging; PD, proton density, ROI, region of interest; AI, artificial intelligence; SR, saturation recovery; T1, longitudinal relaxation time; T2, transverse relaxation time; TE, echo time, TR, repetition time; USG, ultrasonography.

## References

[1] Johnson P.M., Recht M.P., Knoll F. (2020). Improving the Speed of MRI with Artificial Intelligence. Semin. Musculoskelet. Radiol..

[2] Razavian N., Knoll F., Geras K.J. (2020). Artificial Intelligence Explained for Nonexperts. Semin. Musculoskelet. Radiol..

[3] Akazawa M., Hashimoto K. (2021). Artificial intelligence in gynecologic cancers: Current status and future challenges–A systematic review. Artif. Intell. Med..

[4] Maziero D., Straza M.W., Ford J.C., Bovi J.A., Diwanji T., Stoyanova R., Paulson E.S., Mellon E.A. (2021). MR-Guided Radiotherapy for Brain and Spine Tumors. Front. Oncol..

[5] Virostko J. (2020). Quantitative Magnetic Resonance Imaging of the Pancreas of Individuals With Diabetes. Front. Endocrinol..

[6] Río Bártulos C., Senk K., Schumacher M., Plath J., Kaiser N., Bade R., Woetzel J., Wiggermann P. (2022). Assessment of Liver Function With MRI: Where Do We Stand?. Front. Med..

[7] Mori S., Aggarwal M. (2014). In vivo magnetic resonance imaging of the human limbic white matter. Front. Aging Neurosci..

[8] Alexander A.L., Hurley S.A., Samsonov A.A., Adluru N., Hosseinbor A.P., Mossahebi P., Trompdo P.M., Zakszewski E., Field A.S. (2011). Characterization of cerebral white matter properties using quantitative magnetic resonance imaging stains. Brain Connect.

[9] Baudrexel S., Klein J.C., Deichmann R., Hilker R. (2010). Innovative MRT-Verfahren bei idiopathischem Parkinson-Syndrom [Innovative MRI techniques in Parkinson’s disease]. Nervenarzt.

[10] Knight M.J., McCann B., Kauppinen R.A., Coulthard E.J. (2016). Magnetic Resonance Imaging to Detect Early Molecular and Cellular Changes in Alzheimer’s Disease. Front. Aging Neurosci..

[11] Whiting P., Gupta R., Burch J., Mota R.E., Wright K., Marson A., Weishmann U., Haycox A., Kleijnen J., Forbes C. (2006). A systematic review of the effectiveness and cost-effectiveness of neuroimaging assessments used to visualise the seizure focus in people with refractory epilepsy being considered for surgery. Health Technol. Assess..

[12] Wolf M., de Boer A., Sharma K., Boor P., Leiner T., Sunder-Plassmann G., Moser E., Caroli A., Jerome N.P. (2018). Magnetic resonance imaging T1- and T2-mapping to assess renal structure and function: A systematic review and statement paper. Nephrol Dial. Transplant..

[13] MacIver C.L., Tax C.M.W., Jones D.K., Peall K.J. (2022). Structural magnetic resonance imaging in dystonia: A systematic review of methodological approaches and findings. Eur. J. Neurol..

[14] Lazari A., Lipp I. (2021). Can MRI measure myelin? Systematic review, qualitative assessment, and meta-analysis of studies validating microstructural imaging with myelin histology. Neuroimage.

[15] Walecki J., Ziemiański A. (1998). Rezonans Magnetyczny i Tomografia Komputerowa w Praktyce Klinicznej.

[16] Mirhej M.E. (1965). Proton Spin relaxation by paramagnetic molecular oxygen. Can. J. Chem..

[17] Truszkiewicz A., Aebisher D., Żyła G., Sobczak J., Wojtas Ł., Bartusik-Aebisher D. (2021). Wpływ lepkości na pomiar czasu relaksacji podłużnej w diagnostyce z wykorzystaniem MR–Badania wstępne. Inżynier i Fizyk Medyczny.

[18] Deistung A., Schäfer A., Schweser F., Biedermann U., Turner R., Reichenbach J.R. (2013). Toward in vivo histology: A comparison of quantitative susceptibility mapping (QSM) with magnitude-, phase-,and r2-imaging at ultra-high magnetic field strength. Neuroimage.

[19] Hosny A., Parmar C., Quackenbush J., Schwartz L.H., Aerts H.J.W.L. (2018). Artificial intelligence in radiology. Nat. Rev. Cancer.

[20] Zhou L.Q., Wang J.Y., Yu S.Y., Wu G.G., Wei Q., Deng Y.B., Wu X.L., Cui X.W., Dietrich C.F. (2019). Artificial intelligence in medical imaging of the liver. World J. Gastroenterol..

[21] Alsaaidah B., Al-Hadidi M.R., Al-Nsour H., Masadeh R., AlZubi N. (2022). Comprehensive Survey of Machine Learning Systems for COVID-19 Detection. J. Imaging.

[22] Liu H., Chen Y., Zhang Y., Wang L., Luo R., Wu H., Wu C., Zhang H., Tan W., Yin H. (2021). A deep learning model integrating mammography and clinical factors facilitates the malignancy prediction of BI-RADS 4 microcalcifications in breast cancer screening. Eur. Radiol..

[23] Abdelrahman L., Al Ghamdi M., Collado-Mesa F., Abdel-Mottaleb M. (2021). Convolutional neural networks for breast cancer detection in mammography: A survey. Comput. Biol. Med..

[24] Boumaraf S., Liu X., Ferkous C., Ma X. (2020). A New Computer-Aided Diagnosis System with Modified Genetic Feature Selection for BI-RADS Classification of Breast Masses in Mammograms. BioMed Res. Int..

[25] Tsai K.J., Chou M.C., Li H.M., Liu S.T., Hsu J.H., Yeh W.C., Hung C.M., Yeh C.Y., Hwang S.H. (2022). A High-Performance Deep Neural Network Model for BI-RADS Classification of Screening Mammography. Sensors.

[26] Abdelhafiz D., Yang C., Ammar R., Nabavi S. (2019). Deep convolutional neural networks for mammography: Advances, challenges and applications. BMC Bioinform..

[27] Zou L., Yu S., Meng T., Zhang Z., Liang X., Xie Y. (2019). A Technical Review of Convolutional Neural Network-Based Mammographic Breast Cancer Diagnosis. Comput. Math. Methods Med..

[28] Winther H., Hundt C., Ringe K.I., Wacker F.K., Schmidt B., Jürgens J., Haimerl M., Beyer L.P., Stroszczynski C., Wiggermann P. (2021). A 3D Deep Neural Network for Liver Volumetry in 3T Contrast-Enhanced MRI. Rofo.

[29] Do H.P., Guo Y., Yoon A.J., Nayak K.S. (2020). Accuracy, uncertainty, and adaptability of automatic myocardial ASL segmentation using deep CNN. Magn. Reson. Med..

[30] Sander J., de Vos B.D., Išgum I. (2020). Automatic segmentation with detection of local segmentation failures in cardiac MRI. Sci. Rep..

[31] Leiner T., Rueckert D., Suinesiaputra A., Baeßler B., Nezafat R., Išgum I., Young A.A. (2019). Machine learning in cardiovascular magnetic resonance: Basic concepts and applications. J. Cardiovasc. Magn. Reson..

[32] Bratt A., Kim J., Pollie M., Beecy A.N., Tehrani N.H., Codella N., Perez-Johnston R., Palumbo M.C., Alakbarli J., Colizza W. (2019). Machine learning derived segmentation of phase velocity encoded cardiovascular magnetic resonance for fully automated aortic flow quantification. J. Cardiovasc. Magn. Reson..

[33] Wang W., Jiang R., Cui N., Li Q., Yuan F., Xiao Z. (2022). Semi-supervised vision transformer with adaptive token sampling for breast cancer classification. Front. Pharmacol..

[34] Markou M., Singh S. (2006). A Neural Network-Based Novelty Detector for Image Sequence Analysis. IEEE Trans. Pattern Anal. Mach. Intell..

[35] Saeedinia S.A., Jahed-Motlagh M.R., Tafakhori A., Kasabov N. (2021). Design of MRI structured spiking neural networks and learning algorithms for personalized modelling, analysis, and prediction of EEG signals. Sci. Rep..

[36] Chartrand G., Cheng P.M., Vorontsov E., Drozdzal M., Turcotte S., Pal C.J., Kadoury S., Tang A. (2017). Deep learning: A primer for radiologists. RadioGraphics.

[37] Lundervold A.S., Lundervold A. (2019). An overview of deep learning in medical imaging focusing on MRI. Z. Med. Phys..

[38] Maier A., Syben C., Lasser T., Riess C. (2019). A gentle introduction to deep learning in medical image processing. Z/ Med/ Pay/.

[39] Moawad A.W., Fuentes D.T., ElBanan M.G., Shalaby A., Guccione J., Kamel S., Jensen C., Elsayes K. (2022). Artificial intelligence in diagnostic radiology: Where do we stand, challenges, and opportunities. J. Comput. Assist. Tomogr..

[40] Donoho D.L. (2006). Compressed sensing. IEEE Trans. Inf. Theory.

[41] Lustig M., Donoho D., Pauly J.M. (2007). Sparse MRI: The application of compressed sensing for rapid MR imaging. Magn. Reson. Med..

[42] Mohamed A.A., Berg W.A., Peng H., Luo Y., Jankowitz R.C., Wu S. (2018). A deep learning method for classifying mammographic breast density categories. Med. Phys..

[43] Ruck L., Mennecke A., Wilferth T., Lachner S., Müller M., Egger N., Doerfler A., Uder M., Nagel A.M. (2023). Influence of image contrasts and reconstruction methods on the classification of multiple sclerosis-like lesions in simulated sodium magnetic resonance imaging. Magn Reson Med..

[44] Hassan T.M., Elmogy M., Sallam E.S. (2017). Diagnosis of Focal Liver Diseases Based on Deep Learning Technique for Ultrasound Images. Arab. J. Sci. Eng..

[45] Guo L.H., Wang D., Qian Y.Y., Zheng X., Zhao C.K., Li X.L., Bo X.W., Yue W.W., Zhang Q., Shi J. (2018). A two-stage multi-view learning framework based computer-aided diagnosis of liver tumors with contrast enhanced ultrasound images. Clin. Hemorheol. Microcirc..

